# Molecular Diagnosis of Human Papillomavirus: Comparison Between Cervical and Vaginal Sampling

**DOI:** 10.1155/S1064744901000217

**Published:** 2001

**Authors:** Ramzi R. Finan, Noha Irani-Hakime, Hala Tamim, Wassim Y. Almawi

**Affiliations:** 1 Department of Obstetrics and Gynecology St. Georges-Orthodox Hospital Beirut Lebanon; 2 Department of Laboratory Medicine St. Georges-Orthodox Hospital Beirut Lebanon; 3 Department of Epidemiology and Biostatistics Faculty of Health Sciences American University of Beirut Beirut Lebanon; 4 Department of Medical Biochemistry College of Medicine and Medical Sciences Arabian Gulf University P.O. Box 22979 Manama Bahrain

## Abstract

*Background:* Human papillomavirus (HPV) is the most significant cause of cervical cancer. In view of the number of drawbacks associated with endocervical sampling, the gold standard for HPV detection, this study examined the utility and specificity of vaginal sampling as an alternative for endocervical sampling for the routine detection of  HPV.

*Case study:* The study comprised 51 women who tested positive and 54 women who tested negative for endocervical HPV by polymerase chain reaction (PCR), confirmed by histopathology. At the time of specimen collection, both (speculum-assisted) endocervical and vaginal (no speculum) scrapings were isolated from HPV positive and negativewomen, and  HPV DNA was assessed by PCR using the MY09/MY11 primer system;HPV type was identified by hybridization of PCR products with type-specific biotinylated DNA probes. Each participant served as her own control. HPV was detected in vaginal and cervical scrapes from all HPV-positive but not HPV-negative women. In HPV-positive  women the same HPV type was found in vaginal and endocervical scrapings (positive predictive value = 1.0).

*Conclusion:* Correlation between vaginal and endocervical sampling methods was excellent in detecting the presence of HPV DNA and for identifying distinct HPV genotypes. Utilization of vaginal testing for routine HPV detection, and for the long-term follow-up of persistent HPV infection, is therefore recommended.


### Molecular diagnosis of human papillomavirus: comparison between cervical and vaginal sampling

**Ramzi R. Finan[1], Noha Irani-Hakime[2], Hala Tamim[3] and Wassim Y. Almawi[4]**

*[1]Department of Obstetrics and Gynecology, and [2]Department of Laboratory Medicine, St. Georges-Orthodox Hospital, Beirut, Lebanon*
*[3]Department of Epidemiology and Biostatistics, Faculty of Health Sciences, American University of Beirut, Beirut, Lebanon*
*[4]Department of Medical Biochemistry, Arabian Gulf University, Manama, Bahrain*

***Background:*** Human papillomavirus (HPV) is the most significant cause of cervical cancer. In view of the number of drawbacks associated with endocervical sampling, the gold standard for HPV detection, this study examined the utility and specificity of vaginal sampling as an alternative for endocervical sampling for the routine detection of HPV.
***Case study:*** The study comprised 51 women who tested positive and 54 women who tested negative for endocervical HPV by polymerase chain reaction (PCR), confirmed by histopathology. At the time of specimen collection, both (speculum-assisted) endocervical and vaginal (no speculum) scrapings were isolated from HPV-positive and negative women, and HPV DNA was assessed by PCR using the MY09/MY11 primer system; HPV type was identified by hybridization of PCR products with type-specific biotinylated DNA probes. Each participant served as her own control. HPV was detected in vaginal and cervical scrapes from all HPV-positive but not HPV-negative women. In HPV-positive women the same HPV type was found in vaginal and endocervical scrapings (positive predictive value = 1.0).
***Conclusion:*** Correlation between vaginal and endocervical sampling methods was excellent in detecting the presence of HPV DNA and for identifying distinct HPV genotypes. Utilization of vaginal testing for routine HPV detection, and for the long-term follow-up of persistent HPV infection, is therefore recommended.

Key words: HUMAN PAPILLOMAVIRUS, PCR, VAGINAL SCRAPES, PAP SMEAR

### INTRODUCTION

Human papillomavirus (HPV) is the most significant sexually transmitted causative agent of cervical intraepithelial neoplasia (CIN)[1,2], of which more than 100 types have been characterized[3]. Traditional screening for HPV infection relied on the Pap smear. Although the Pap smear has been widely adopted in the initial screening for CIN, the high number of false negatives and positives associated with it[4,5], coupled with the need for highly sensitive methods for detecting HPV infection in high-risk women and women highly suspected of infection, has necessitated a search for alternative methods for HPV screening to assess cervical neoplasia. Identification of HPV DNA by molecular biology tools, in particular polymerase chain reaction (PCR), has been reported by several investigators to be more sensitive than cytology in the identification, and also in the monitoring of the progression, of CIN disease[5,6].

Correspondence to: Dr Wassim Y. Almawi, PhD, Department of Medical Biochemistry, College of Medicine and Medical Sciences, Arabian Gulf University, P.O. Box 22979, Manama, Bahrain. E-mail: wassim@agu.edu.bh

Several methods have been used in detecting HPV infection, including PCR[4,5,7], which relies on isolating endocervical epithelial cells by a gynecologist using a speculum and subsequent DNA analysis. However, this technique is associated with drawbacks with regard to patient compliance, its invasive nature, and the poor yield of tissue for repetitive sampling[8], prompting investigation into alternative methods, including vaginal testing, that yield sufficient cells without inconveniencing the patient[8,9]. Varied concordance rates have been obtained between speculum-assisted endocervical sampling and other collection methods[1,8,9], highlighting the need for refinements in specimen collection and further manipulation. Here we extend earlier findings by demonstrating the usefulness and sensitivity of vaginal testing in detecting HPV genotypes. The presence of HPV and β-actin DNA sequences in endocervical and vaginal scrapes was assessed in HPV-positive and HPV-negative women; complete concordance between these two sampling methods was obtained.

### SUBJECTS AND METHODS

### Study subjects

The study involved 105 women, of whom 51 were followed because of confirmed endocervical HPV infection, the remaining 54 women being free of HPV as assessed by immunohistochemistry and confirmed by PCR. For the purpose of this study both endocervical and vaginal specimens were collected at the same time to minimize variations in viral load, and hence sensitivity, between visits. All participants were asked to complete a standard questionnaire that details age, marital status, smoking, pregnancies, number of sexual partners, concurrent infections and results of Pap smear (and date taken). After the purpose and implications of the findings were explained to all participants, and after all institutional ethics requirements were met, each participant was asked to sign an informed confidential consent form agreeing to participate in the study.

### Specimen collection

Endocervical scrapes were collected by speculum-assisted spatula of Ayre after removing the mucus, and were placed in balanced saline solution. Vaginal scrapes were collected by introduction of the sterile spatula of Ayre after separating the labia minora (to visualize the introitus), followed by intra-vaginal 180° rotation to collect scrapings, which were immediately placed in balanced saline solution. Total genomic DNA was extracted by the phenol–chloroform method, as is standard in our laboratory.

### HPV DNA amplification

DNA samples were amplified with the L1 consensus HPV primers MY09/MY11[7]. The primer sequences for MY09 are 5'-CGT CC(A,C) A(A,G)(A,G) GGA (A,T)AC TGA TC-3', and for MY11, 5'-GC(A,C) CAG GG(A,T) CAT AA(C,T) AAT GG-3'. Amplification of the 'house-keeping' gene, β-actin, was performed in parallel on every sample in order to control for DNA integrity and to rule out the presence of inhibitors of amplification. The primer sequences for β-actin were: forward, 5'-GTG GGG CGC CCC AGG CAC CA-3', and reverse, 5'-CTC CTT AAT GTC ACG CAC GAT TTC-3'. PCR conditions comprised an initial denaturation for 2.5 min at 96°C, 41 cycles of denaturation for 30 s at 92°C, annealing for 30 s at 56°C, and extension for 45 s at 72°C. PCR products were electrophoresed on ethidium bromide-stained agarose gel (2%), and visualized under UV-transillumination. HPV genotypes were determined by hybridization with genotype-specific biotinylated DNA probes, and visualized by DNA enzyme immunoassay (DEIA) as per manufacturer's specification (DiSorin, Salluggia, Italy).

### RESULTS

### Patient profile

A total of 105 women participated in the study, of whom 51 were HPV-positive and 54 were HPV-negative, as shown by the 'gold standard', endocervical HPV detection. While there was no statistically significant difference between HPV-positive and HPV-negative women with respect to age (32.9 ± 8.3 and 32.5 ± 6.9 years, respectively, $p = 0.93$), those who had had at least one pregnancy (36/51 and 33/54, respectively, $p = 0.404$),

**Table 1** Profile of study group

| *Variable* | *Total* | *HPV +ve* | *HPV –ve* | p *value* |
|---|---|---|---|---|
| *n* | 105 | 51 | 54 | |
| Age ± SD (years) | 32.7 ± 7.6 | 32.9 ± 8.3 | 32.5 ± 6.9 | 0.93* |
| Range | 21–56 | 21–56 | 21–52 | |
| Smokers (*n* (%)) | 48 (45.7) | 28 (54.9) | 20 (37.0) | 0.078** |
| Women who had had at least one pregnancy (*n* (%)) | 69 (65.7) | 36 (70.6) | 33 (61.1) | 0.404** |
| Women who had had multiple partners (*n* (%)) | 57 (54.3) | 35 (68.6) | 22 (40.7) | 0.073** |
| Abnormal Pap smear (*n* (%)) | 47 (44.8) | 41 (80.4) | 6 (11.1) | <0.001** |

*Student's *t* test (two-tailed); **Pearson's $\chi^2$ test; HPV, human papillomavirus smoking (28/51 and 20/54, respectively, $p = 0.078$), or to those with more than one male partner (35/51 and 22/54, respectively, $p = 0.073$), a higher prevalence of abnormal Pap smear was seen in HPV-positive compared with HPV-negative women (41/51 and 6/54, respectively, $p < 0.001$) (Table 1).

**Table 2** HPV DNA in vaginal and cervical scrapes of HPV-positive ($n=51$) and HPV-negative ($n=54$) women; sensitivity and specificity, as well as positive and negative predictive values, were 1.0

| | *Cervical scrapings* | |
|---|---|---|
| *Vaginal scrapings* | *Positive* | *Negative* |
| Positive | 51/51 | 0/54 |
| Negative | 0/51 | 54/54 |

### Detection of HPV DNA in cervical and vaginal scrapings

All endocervical and vaginal samples obtained from study participants were initially tested for the presence of β-actin sequences. All were positive for β-actin, and hence were subjected to PCR-based amplification of HPV consensus sequences. Vaginal testing detected HPV DNA in all specimens of the 51 HPV-positive cervical scrapes and none in the 54 HPV-negative cervical scrapes (sensitivity, 1.0; specificity, 1.0) (Table 2). Insofar as all specimens screened were positive for β-actin, this ruled out the possibility of loss of DNA integrity in samples, and/or nonspecific inhibition of amplification, demonstrating that all HPV-positive and all HPV-negative vaginal specimens were truly positive and negative, respectively, when determined by cervical scrapes (positive and negative predictive values = 1.0). Furthermore HPV type(s) detected in vaginal scrapes was/were identical to that/those found in cervical scrapes (data not shown).

### DISCUSSION

In comparing vaginal with endocervical scrapes as the specimen of choice, excellent correlation was obtained in detecting HPV DNA. In addition, HPV genotype analysis revealed that both sampling methods were concordant for 100% of sample pairs. The sampling device used in this study, the spatula of Ayre, gave both consistent and reproducible results, especially in women who were retested (data not shown), in agreement with reports documenting its superiority as the means of collection rather than self-collected devices including cotton swabs, brushes, and lavages[4,8–10]. The use of the latter were associated with lower rates of HPV detection.

Vaginal testing afforded a significant improvement in sensitivity and specificity over previous reports, which showed a correlation of 80–90% between vaginal and cervical sampling methods[8,9]. In the quoted studies failure to produce the 100% correlation between cervical and vaginal specimen was most probably the result of sampling problems[4,9,10]. In this study we opted to use vaginal scrapes (vs tampons or lavages), which produced sufficient quantities of cells for analysis[8,9]. Also, we routinely tested for the expression of the housekeeping gene, β-actin, as control for the presence of genomic DNA. In the present study all samples assayed were positive for β-actin, thereby ruling out the possibility that absence of HPV amplifiable products was not due to specimen degradation or to the presence of inhibitors of amplification, further confirming the consistency and efficacy of this sampling technique[4,8].

HPV DNA was amplified by PCR using the MY09/MY11 primer pair, described as the primer pair of choice in detecting HPV DNA[7]. All cervical and vaginal scrapes were collected at the same time to rule out the possibility of variations in viral load, and hence sensitivity, between the cervix and vagina[6,8]. A limitation of our study that could not be avoided was that vaginal sampling required a gynecologist/technologist for specimen collection[1,8]. Nevertheless, it was less invasive as compared to endocervical sampling, since it did not require insertion of a speculum for sample collection. Furthermore it was well tolerated, and none of the participants experienced any discomfort during specimen collection. This demonstrates the usefulness and sensitivity of vaginal sampling as an equally sensitive but less invasive procedure for detecting the presence of HPV genotypes.

### CONCLUSIONS

Vaginal testing is an easy yet highly valid method for detecting HPV DNA. Correlation between vaginal and cervical sampling methods was excellent for the presence of HPV DNA and for identifying distinct HPV genotypes. It is recommended that vaginal sampling should be used in initial HPV screening, especially in high-risk/suspicious women with cytomorphologically normal smears, and/or where cervical testing may not be feasible, as in virgin girls (insistence on hymen integrity owing to social/cultural considerations) who may have contracted HPV through routes other than intra-vaginal intercourse (deep petting, anal intercourse, etc.). Furthermore, vaginal screening may be of use in the long-term follow-up of HPV-infected women for viral persistence, and hence monitoring CIN disease status[4].

### REFERENCES

1. Duggan MA, McGregor SE, Stuart GC, *et al.* The HPV determinants of CIN I. *Eur J Gynaecol Oncol* 1997;18:117–23
2. Strand A, Rylander E. Human papillomavirus. Subclinical and atypical manifestations. *Dermatol Clin* 1998;16:817–22
3. Dillner J, Meijer CJ, von Krogh G, Horenblas S. Epidemiology of human papillomavirus infection. *Scand J Urol Nephrol Suppl* 2000;205:194–200
4. Lytwyn A, Sellors JW, Mahony JB, *et al.* Comparison of human papillomavirus DNA testing and repeat Papanicolaou test in women with low-grade cervical abnormalities: a randomized trial. *Can Med Assoc J* 2000;163:701–7
5. Rozendaal L, Walboomers JM, van der Linden JC, *et al.* PCR-based high-risk HPV test in cervical cancer screening gives objective assessment of women with cytomorphologically normal cervical smears. *Int J Cancer* 1996;68:766–9
6. Gjoen K, Sauer T, Olsen AO, Orstavik I. Correlation between polymerase chain reaction and cervical cytology for detection of human papillomavirus infection in women with and without dysplasia. *Acta Pathol Microbiol Immunol Scand* 1997;105:71–5
7. Harnish DG, Belland LM, Scheid EE, Rohan TE. Evaluation of human papillomavirus – consensus primers for HPV detection by the polymerase chain reaction. *Mol Cell Probes* 1999;13:9–21
8. Coutlee F, Hankins C, Lapointe N and The Canadian Women's HIV Study Group. Comparison between vaginal tampon and cervicovaginal lavage specimen collection for detection of human papillomavirus DNA by the polymerase chain reaction. *J Med Virol* 1997;51:42–7
9. Sellors JW, Lorincz AT, Mahony JB, *et al.* Comparison of self-collected vaginal, vulvar and urine samples with physician-collected cervical samples for human papillomavirus testing to detect high-grade squamous intraepithelial lesions. *Can Med Assoc J* 2000;163:513–18
10. Peng HQ, Roth P, Causs D, Rawls W. Comparison of the cytobrush and cotton swabs in sampling cervical cells for filter *in situ* hybridization detection of human papillomavirus types 16 and 18 DNA. *Acta Cytologica* 1988;32:311–13

Received 11/28/00; Accepted 04/03/01
